# The climate comfort assessment for tourism purposes in Borobudur Temple Indonesia

**DOI:** 10.1016/j.heliyon.2020.e05828

**Published:** 2020-12-28

**Authors:** Nur Aini Iswati Hasanah, Devianti Maryetnowati, Fauziah Nurchaulia Edelweis, Fadhila Indriyani, Qorry Nugrahayu

**Affiliations:** Department of Environmental Engineering, Universitas Islam Indonesia (UII), Yogyakarta 55584, Indonesia

**Keywords:** Climatic comfort, Holiday climate index, Temperature humidity index, Tourism, Tourism climate index, World heritage

## Abstract

Included on the World Heritage List in 1991, the Borobudur Temple Compounds have been attracting vast numbers of tourists. To increase the number of incoming tourists even further, tourism climate comfort is one of the key elements that must be considered, as it can affect tourist visiting patterns, as well as tourists' preferences. This study analyzes the level of tourism climate comfort using three different methods, i.e., Tourism Climate Index (TCI), Temperature Humidity Index (THI) and Holiday Climate Index (HCI). It further assesses their sensitivities to observe tourist visitation in Borobudur Temple. The climate data for the timeframe 2010–2019 is obtained from the Automatic Weather Station (AWS) Borobudur. Based on the TCI, we notice that the comfortable months were June to August. By comparison, the THI results showed that the comfortable period began in July and ended in August. Meanwhile, the HCI results showed that the comfortable months were July to October. All monthly indexes obtained by means of the three methods are then evaluated in view of the monthly visitation data. This optimized HCI index demonstrates a high correlation with tourist visitation at Borobudur Temple, especially foreign tourist that more sensitive to the climate. Hence, relative humidity and rainfall have high correlation with the HCI climatic comfort level at Borobudur Temple. This study improves our understanding of the climatic index correlation with the number of visitors at Borobudur Temple and can help advertisers and local authorities make pre-trip climate comfort information and more informed decisions in terms of tourism policies.

## 1 Introduction

Climate comfort is characterized as the level of satisfaction with climatic conditions in the environment in which people take part in tourist activities. While the comfort conditions vary according to the type of tourism discussed, the presentation of certain threshold values and directories can ensure a clearer picture of the comfort level associated with the climatic conditions of the environment (Öztürk and Göral, 2018). Several climate comfort indexes have been developed to highlight the diverse nature of climate resources for tourism (Anđelković et al., 2016).

Tourism Climate Index (TCI) is a quantitative assessment of the tourism climate which merges tourism-related climate factors into a single index. This is one of the most cited and widely acknowledged climate indexes, deriving from the human comfort impression (Méndez-lázaro et al., 2014). A TCI comprises thorough metrics that combine all three vital climate factors related to tourism: thermal comfort; physical aspects, such as rain and wind; and the aesthetics of sunshine or cloudiness. Matusiak (2010) defines the term thermal comfort as a satisfaction state taking of the environment thermal conditions resulting from the thermal effects of the environment; hence, thermal comfort creates a physiological impact. Physical aspects create physical impact that conforming to the comfort level obligatory for the tourist activities entail physical effort. Meanwhile, the aesthetic component of climatic suitability for tourism is widely held as a nature-related form of psychological impact (Fichett et al., 2016; Kovács and Unger, 2014). Furthermore, a TCI uses climate variables that are easy to obtain from weather stations or climate models, which in turn simplifies data provision (Qing-ping & Fang-lei, 2019).

Even though complex energy balance models ensure an overall outlook of human bio climate, none can match the potential that the Temperature Humidity Index (THI) has in giving a general approach of stress changes and replicate the thermal sensation of a human being. This representation of the bioclimatic conditions in the area over time (Ciobotaru et al., 2018) and remains the most frequently used bio climate index in the tropics, particularly outdoors. More specifically, THI is an alteration of equivalent temperatures. For calculation, it uses air temperature and relative humidity to associate expected conditions of the wet and dry bulb temperatures (Emmanuel, 2005).

Holiday Climate Index (HCI) had been developed to assess the climatic suitability of certain destinations for tourism. The word ‘holiday’ was chosen to highlight what the index was aimed at (i.e., leisure tourism). Tourism is much broader by definition. According to United Nations (2010), tourism is a social, cultural and economic phenomenon, which assumes the movement of people to countries or places outside their usual environment for personal or business/professional purposes. An important advantage of the HCI is that its variable rating scales and the component weighting system draw upon the extensive literature analyzing tourists' stated climatic preferences over the last decade (Scott et al., 2016). Even though this index more complex compare to TCI and THI, Hejazizadeh et al. (2019) stated that this index is empirically confirmed by the characteristics of the tourist market.

The goals of this paper are to analyze the correlation between the tourism climate comfort level obtained with the help of three different methods, i.e., TCI, THI, and HCI; and to determine the most sensitive index for analyzing tourism climate comfort level based on the example of the Borobudur Temple. We predict that the more complex the method, the more accurate the results of the climate comfort analysis. However, we want to verify this hypothesis by means of this study. The comparison of the three climate comfort indexes will also reveal how the differential treatment of individual variables (e.g., overriding variables) affects rating outcomes. Therefore, another goal of this paper is to identify the overriding variables from the indexes.

## 2 Data and methods

### 2.1 Study area

Borobudur Temple Compounds is a Buddhist temple complex which was included on the World Heritage list in 1991. As a result, it has become a famous tourist destination in Indonesia and has strong international appeal (Hermawan et al., 2019). More specifically, it is one of the 10 most visited destinations in Indonesia, its unique cultural value bringing an important economic advantage to the area (Hindami et al., 2018).

**Figure 1:**
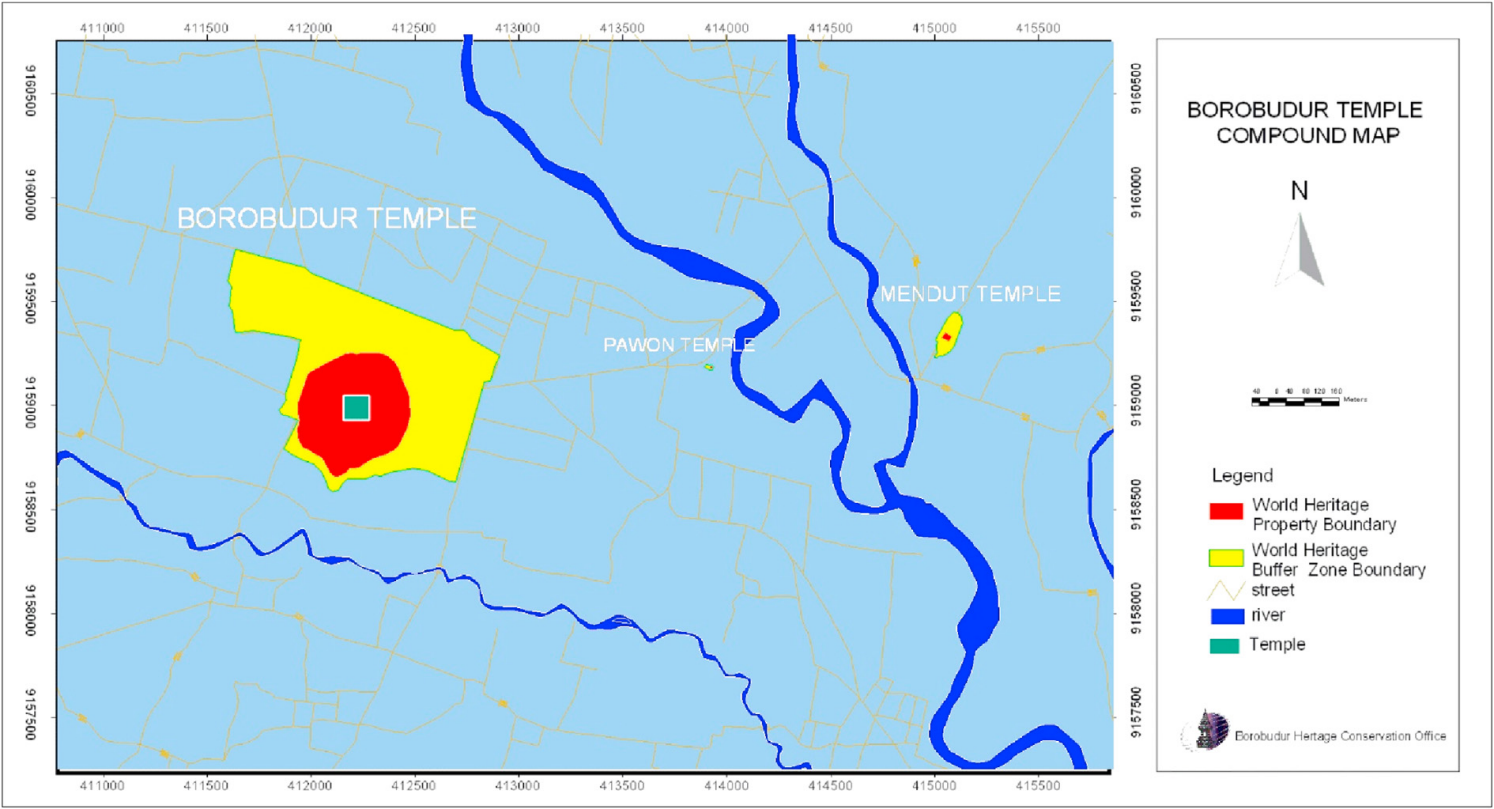
Borobudur Temple compound map.

The Borobudur Temple Compounds (Figure 1) encompasses three stone temples, i.e., Mendut, Pawon and Borobudur. Borobudur is the main one, as it is the largest of the three. It lies about 40 km north of Jogjakarta city, in the central part of Java Island (S 7^o^36′28.008″, E 7^o^36′28.008″). The Government of Indonesia (GoI) has included Borobudur Temple into the list of 15 excellent tourist destinations, and the initiative to preserve the precious cultural heritage of Borobudur has drawn national and international attention (Gunarto, 2017; Hermawan et al., 2019).

The cultural landscape of Borobudur, which is a combination of local culture and natural resources is extremely beautiful (Susilo and Suroso, 2014). The natural resources that exist in the vicinity of this site vastly increase the value of Borobudur's heritage. The Borobudur Temple stands on a hill that rises approximately 15 m above surrounding hills, mountains and large rivers. Despite the rapid developments currently taking place in the Borobudur area, most of the region consists of agricultural land planted with paddy, which has a significant influence on the local climate (Ekarini, 2017).

The geographical location imposes a tropical climate. However, the area is also subject to frequent rainfall by comparison with other regions in Central Java and the Special Region of Yogyakarta (Sudibyo, 1996).

### 2.2 Data collection

We used daily climatic datasets from January 1, 2010 to December 31, 2019. This data was obtained from the Borobudur automatic weather station (7.607999, 110.2045), which operated by the Meteorological, Climatological and Geophysical Agency (BMKG) Semarang. The data included average air temperature, maximum air temperature, average relative humidity, minimum relative humidity, rainfall, wind speed and sun hour. Monthly tourist visitation data for the year 2013–2019 was collected from the Borobudur Conservation Office and the Statistics of Magelang Regency.

### 2.3 Tourism Climate Index (TCI)

Mieczkowski (1985) designed the TCI method with the following equation:

TCI = 2[(4CID) + CIA + (2P) + (2S) + W]

Where CID = comfort index during the day (°C), CIA = daily comfort index (°C), P = rating based on total monthly rainfall (mm), S = rating based on sun hour (hours/day) and W = rating based on wind speed (km/hour). The values of the CID and CIA variables follow the rating scheme in Table 1, being based on the effective temperature, while the other parameters are based on its value. The CID value is the result of a combination of maximum air temperature with minimum relative humidity. CIA is the result of a combination of average air temperature with average relative humidity using a psychometric chart. The TCI values that have been obtained show the level of tourist comfort based on the values in Table 2.

**Table 1:** A variable rating scheme (sub-index) in TCI.

Rating	Effective temperature (^o^C)	Monthly rainfall (mm)	Sun hour (hour)	Wind speed (km/hour)
5.0	20–26	0–14.9	>10	<2,88
4.5	19	15–29.9	9	2,88–5,75
4.5	27	16–29.9	9	2,88–5,76
4.0	18	30–44.9	8	5,76–9,03
4.0	28	31–44.9	8	5,76–9,04
3.5	17	45–59.9	7	9,04–12,23
3.5	29	46–59.9	7	9,04–12,24
3.0	16	60–74.9	6	12,24–19,79
3.0	30	61–74.9	6	12,24–19,80
2.5	10–15	75–89.9	5	19,8–24,29
2.5	31	76–89.9	5	19,8–24,30
2.0	5–9	90–104.9	4	24,30–28,79
2.0	32	91–104.9	4	24,30–28,80
1.5	0–4	105.0–119.9	3	28,8–38,52
1.5	33	105.0–119.9	3	28,8–38,53
1.0	(-5) - (-1)	120–134.9	2	-
1.0	34	121–134.9	2	-
0.5	35	135–149.9	1	-
0.25	-	-	-	-
0	(-10) - (-11)	>150	<1	>38,52
0	>36	>151	<2	>38,53
-1.0	(-15) - (-11)	-	-	-
-2.0	(-20) - (-16)	-	-	-
-3.0	< (-20)	-	-	-

**Table 2:** TCI climate comfort levels for tourism.

Index value	Descriptions of climate comfort levels
90–100	ideal
80–89	excellent
70–79	very good
60–69	good
50–59	acceptable
40–49	marginal
30–39	unfavorable
20–29	very unfavorable
10–19	extremely unfavorable
-3–9	impossible

### 2.4 Temperature Humidity Index (THI)

Nieuwolt (1977) developed THI in the form of an equation as follows:

THI = (0.8T) + {(RHT)/500}

Where T is the air temperature (°C) and RH is the relative humidity (%). The THI value obtained shows the level of tourism climate comfort based on the values presented in Table 3. The latter are the result of an empirical test on human subjects, developed by Emmanuel (2005) and carried out in the tropics.

**Table 3:** THI climate comfort levels for tourism.

Index value	Descriptions of climate comfort levels
21–24	comfortable
25–26	half uncomfortable
>26	uncomfortable

### 2.5 Holiday Climate Index (HCI)

Mieczkowski (1985) designed the HCI method with the following equation:

HCI = 4(TC) + 2(A) + (3(P) + W)

Where TC is the thermal comfort rating calculated using the following equation:

TC = (0. 8T + (RHT)/500) × 4

Where T is the maximum daily air temperature (°C) and RH is the relative humidity (%). A is an aesthetic factor related to the rating based on cloud cover (%). The physical factors are based on P and W, where P = rating based on total rainfall (mm) and W = rating based on wind speed (km/hour). The ratings are presented in Table 4 (Tang, 2013). The HCI value obtained shows the level of comfort of the tour based on the rating value in Table 5.

**Table 4:** A variable rating scheme (sub-index) in HCI.

Rating	Air temperature (°C)	Wind speed (km/hour)	Cloud cover (%)	Rainfall (mm/day)
10	23–35	1–9	11–20	0.00
9	20-22 or 26	10–19	1-10 or 21-30	<3.00
8	27–28	0 or 20-29	0 or 31-40	3.00–5.99
7	18-19 or 29-30	-	41–50	-
6	15-17 or 31-32	30–39	51–60	-
5	11-14 or 33-34	-	61–70	6.00–8.99
4	7-10 or 35-36	-	71–80	-
3	0–6	40–49	81–90	-
2	37-39 or (-1)-(-5)	-	91–99	9.00–12.00
1	≤-6	-	100	-
0	≥39	50–57	-	>12.00 and ≤25.00
-1	-	-	-	>25.00
-10	-	>70	-	-

**Table 5:** HCI climate comfort levels for tourism.

Index value	Descriptions of climate comfort levels	
90–100	ideal	comfortable
80–89	excellent	
70–79	very good	
60–69	good	
50–59	acceptable	uncomfortable
40–49	marginal	
10–39	unfavorable	
0–9	very unfavorable	

However, cloud cover data is not available at the Borobudur Automatic Weather Station. According to Rangarajan and Swaminathan (1984), cloud cover values can be estimated using the Angstrom-Savinov equation, developed by Kondratyev (1969):

C = (1-(H/H_o_)) / (1-k)

k is a constant defining transmission of solar radiation within the clouds, generally 0.33 in low latitudes. Meanwhile, H/H_o_ is the ratio of measured global solar radiation (H) and extraterrestrial radiation (H_o_), known as a clarity index that shows what percentage of the incoming sky radiation is reflected and highlights changes in the atmospheric conditions of a particular region. The solar radiation data is not available either, but is estimated using Sarkar (2016):

H = 0.6093(S/S_o_) ^0,478^H_o_

S is recorded sun hour (hours), meanwhile S_o_ (day length) and Ho are calculated based on Sarkar (2016):

S_o_= (2/15) ω_s_

H_o_ = 37.6d_r_ [(ω_s_sin *ϕ*sin δ) + (sin ω_s_ cos *ϕ*cos δ)]

d_r_ = (1 + 0.33) cos (0.0172)

ω_s_ = arcos [-tan δ tan *φ*]

δ = 0.409sin (0.0172 J -1.39)

*ϕ* = (πL)/180

J is a Julian date and L is latitude.

### 2.6 Correlation study

A correlation study is used to determine the relationship among indexes and between the index and tourist visitation data. Zero correlation coefficient (r) indicates no relationship between the two variables, and r = 1 or r = −1 indicates a perfect relationship. The strength can be anywhere between 0 and ±1 (Mondal and Mondal, 2016). In this paper, the correlation coefficients are analyzed using Microsoft Excel and will be interpreted based on Table 6, created according to Hinkle et al. (2003). Rather than using other oversimplified rules in method, we opted for the correlation rank, because it is simple and widely used but powerful enough to describe the strength and direction of an association between variables and thus answer the question posed here (Taylor, 1990); this, in turn, has turned into an advantage of the proposed article, as it can be adapted by future researchers.

**Table 6:** Rule of thumb for interpreting the size of a correlation coefficient.

Size of Correlation	Interpretation
0.90 to 1.00 (−0.90 to −1.00)	Very high positive (negative) correlation
0.70 to 0.90 (−0.70 to −0.90)	High positive (negative) correlation
0.50 to 0.70 (−0.50 to −0.70)	Moderate positive (negative) correlation
0.30 to 0.50 (−0.30 to −0.50)	Low positive (negative) correlation
0.00 to 0.30 (0.00 to −0.30)	Negligible correlation

## 3 Result and discussion

### 3.1 Climate characteristic

Over the past 10 years, the Borobudur temples have had minimum, maximum and average temperatures of 21 °C, 33.2 °C and 26.3 °C, respectively. Meanwhile, the average air humidity, rainfall, wind speed and cloud cover are 80.1%, 185 mm/day, 2.65 km/h and 0.626, respectively.

Based on the Mohr classification, the climatic conditions in the Borobudur Temple over the past ten years (2010–2019) showed that there were two humid months (June and September) and two dry months (July to August), while others were eight wet months. Therefore, the climate at Borobudur Temple was included in the class 1 category (Wet).

### 3.2 Tourism climate comfort in Borobudur Temple

In this study, monthly changes in the value of tourism climate comfort 10-year periods have been studied using three indexes, e.g., TCI, THI and HCI. The indexes value is the average of 10-year periods of data in the same month.

**Figure 2:**
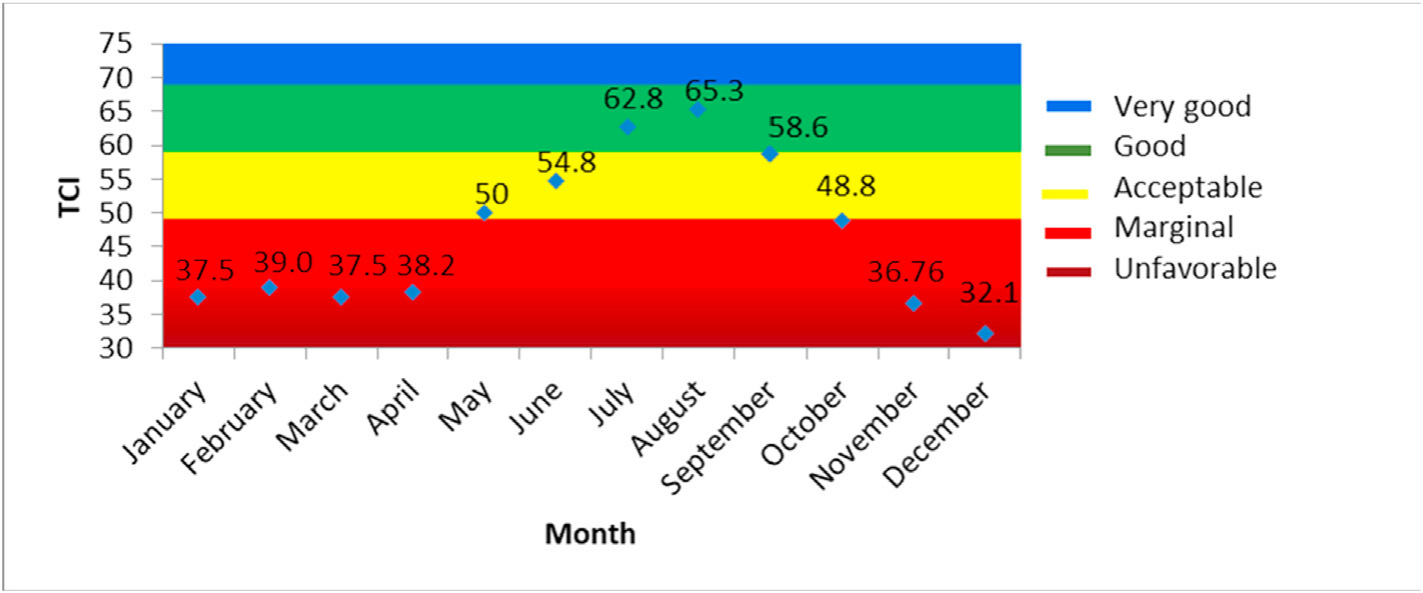
TCI climate comfort level in Borobudur Temple.

Figure 2 shows the climate comfort in Borobudur Temple based on the TCI index. The TCI value was 62.8 in July, and 65.3 in August. The comfortable scale is 60–100, so both months are comfortable. The uncomfortable scale is between -9 to 59. Based on Scott and Mcboyle (2001), these monthly climate comfort values have varied and reached a peak during the dry season. January to April have a level of comfort that is not much different. There was an increase in the level of comfort from May until August, with a peak in August, and then there was a decline from September to December. August was classified as a dry month; this supports dry season peak suggested by the TCI result.

**Figure 3:**
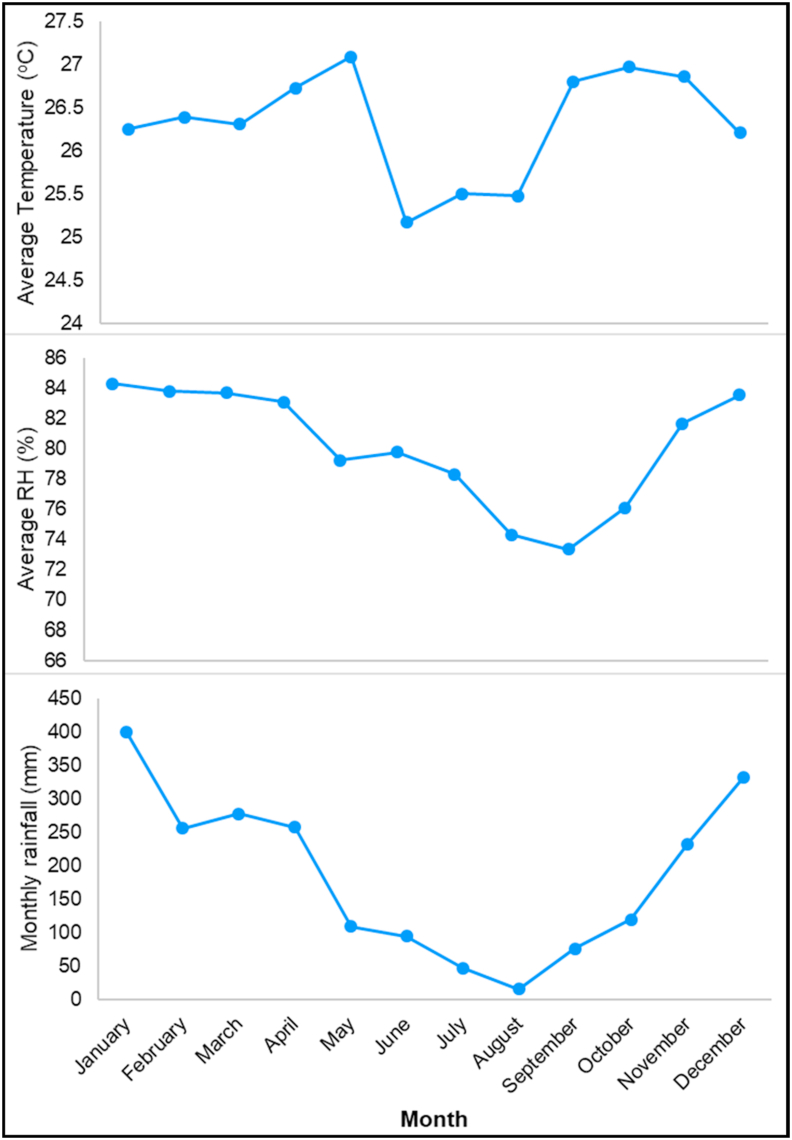
Monthly average temperature, relative humidity, and rainfall.

In August, which was a dry month and served as, we can find relatively low average values in terms of air temperature and relative humidity, and the lowest precipitation rate; this can be seen in Figure 3. The lowest rainfall and relative humidity are likely to occur during the dry season. The relatively low average air temperature also occurs, related to its dry season. According to Maru and Ahmad (2015), high temperatures in Indonesia usually occur during the transition between the dry and wet season. However, based on Roshan and Rousta (2009), in TCI index, the highest rate with a total of 50 belongs to components of temperature and relative humidity. Hence, the trend change in both of these components during the dry months (not only August but also July) could have a major effect on comfortable conditions as described by means of the TCI coefficient, whereby the main components in climate-tourism time oscillations are temperature and humidity.

**Figure 4:**
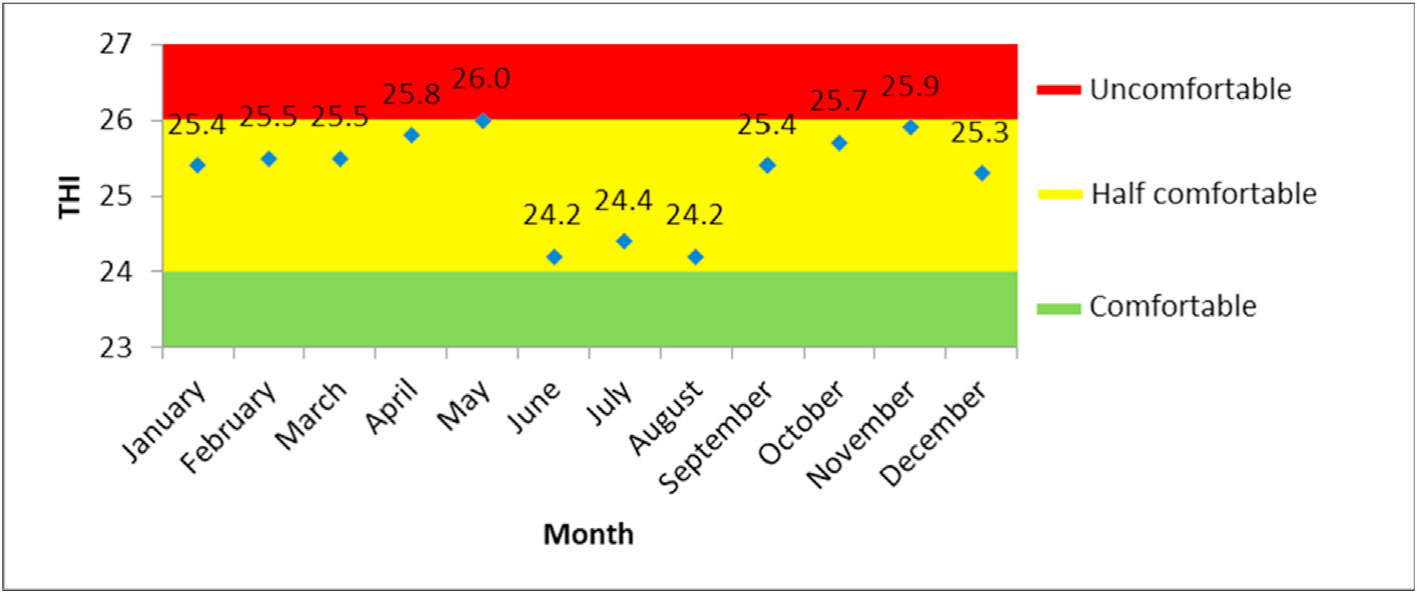
THI climate comfort level in Borobudur Temple.

Based on the results of the THI analysis (Figure 4), from June to August, the climatic conditions in the tourist area of the Borobudur Temple were in the *comfortable* category, with comfort values ranging from 21 to 24. According to Scott and Mcboyle (2001), these monthly climate comfort values are varied and show Bimodal – Shoulder Peaks. From January to May, the comfort value obtained in the THI analysis increased, reaching its first peak in May (first peak); as a result, it is classified in the *medium* category. From June to August, there was a decrease and the value even reached its lowest point, being classified as *comfortable*. From September to November, the value increased again, only to drop in December; yet it is classified as *medium*. The second peak occurred in November.

Hence, based on the THI analysis, the Borobudur offered comfortable climatic conditions in June, including the humid month category; July and August are then included in the dry month category. This result is different from the TCI one, which found that comfortable climate conditions occur only in the dry months. A similar conclusion was also reached by Hassan et al. (2015), because the TCI calculation used other meteorological parameters, which could be more accurate. As such, further analysis is needed.

**Figure 5:**
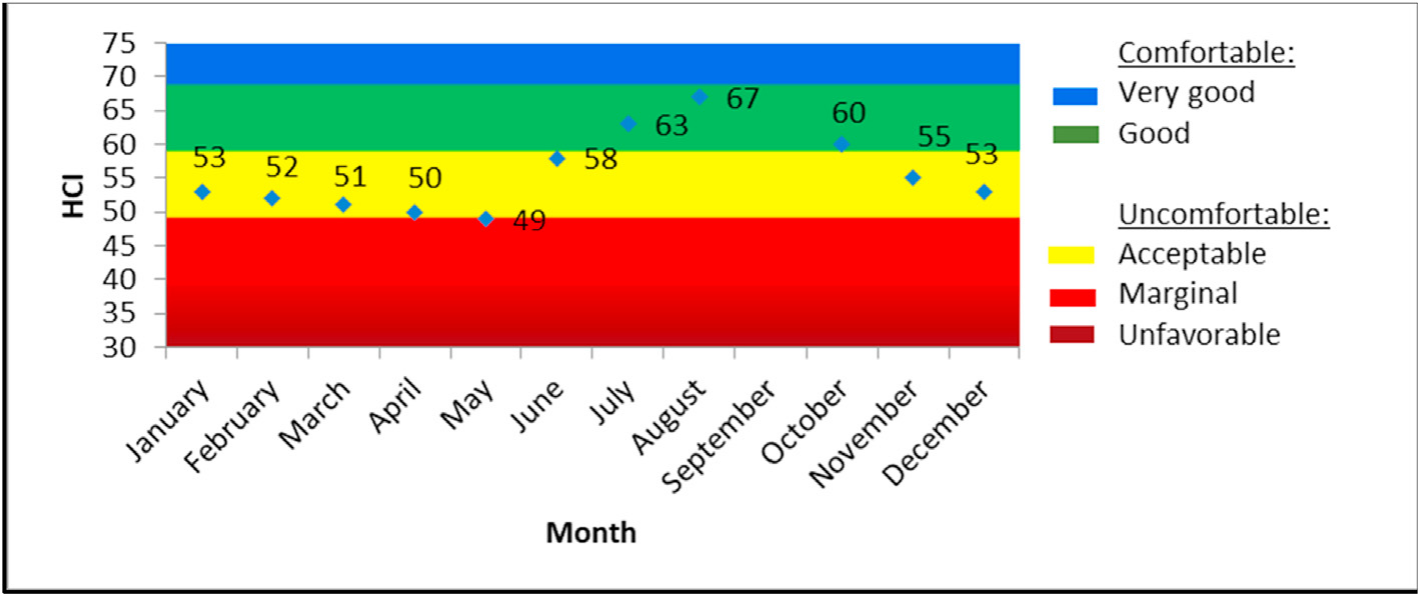
HCI climate comfort level in Borobudur Temple.

Monthly HCI values range from 53 to 70, categorized as acceptable to good. This can be seen in Figure 5. It occurs in several months from June to January. According to Scott and Mcboyle (2001), these monthly climate comfort values fluctuated and showed a dry season peak, similar to TCI. HCI values in the wet months gradually begin to decrease from January to May, and then increase in the dry months, with a peak in September. The peak is situated at 70, then the value drops again from October to December.

Both the TCI and HCI analyses embrace effective temperatures for the thermal aspect. However, the TCI gives half of its total weighting to thermal comfort, making it an essential factor in determining climatic suitability of an urban destination for tourism. By contrast, HCI overcomes the main limitations of the extensively used TCI, as it takes the combined influence of rain and wind into consideration and uses daily data (Scott et al., 2016). The result proves that acceptable to good tourism climate conditions occur in 8 months, categorized in all seasons, i.e., dry, humid and wet.

### 3.3 Correlation between tourism climate comfort indexes in Borobudur Temple

The result showed a similar peak type in TCI and HCI. By comparison, THI reveals a trend that is different from the one suggested by the other two indexes. A correlation study is therefore needed to explore the relationship between the indexes.

Based on the correlation analysis (Table 7), we can state that the TCI and the HCI methods have a high correlation (r = 0.781367). This value surpasses the correlation with other methods, i.e., TCI with THI (r = -0.66479) and THI with HCI (r = -0.51038), both of which are classified as moderate.

**Table 7:** Correlation rank between each climate comfort levels from various indexes.

r		x		
		TCI	THI	HCI
y	TCI	1	-0.66479	0.781367
	THI	-0.66479	1	-0.51038
	HCI	0.781367	-0.51038	1

It is possible for a high correlation to occur between HCI and TCI. According to Rutty et al. (2020), both TCI and HCI employ an additive approach. Thus, each of the sub-indices is weighted to represent the proportional contribution of each climatic variable, with variable weights portrayed through expert judgement (TCI). This also explains why THI has less correlation with the other two indexes.

However, HCI and TCI show different climate comfort levels. The HCI value is always higher than TCI. This is also confirmed in the study carried out by Scott et al. (2016); the TCI yields the highest score when there are more than 10 h of sunshine, and the rating scores become lower as the number of hours of sunshine decreases. In the Borobudur Temple, the maximum monthly average of sun hours is 9.9 h/day. Therefore, the TCI value will be lower than HCI.

### 3.4 Correlation between tourism climate comfort and tourist visitation data in Borobudur Temple

The Borobudur Temple has become increasingly popular since its reopening, not only to domestic visitors but also to international ones. The annual number of visitors reaches 3,694,665. The foreign visitors amount to 2.5% of the total number of international tourists coming to Indonesia, and the temple is the most visited paid-entry cultural site in the country. The top nationalities of foreign tourists visiting Borobudur are Dutch, Japanese, Malaysian, French and German (Cochrane, 2019; Cochrane et al., 2019).

**Figure 6:**
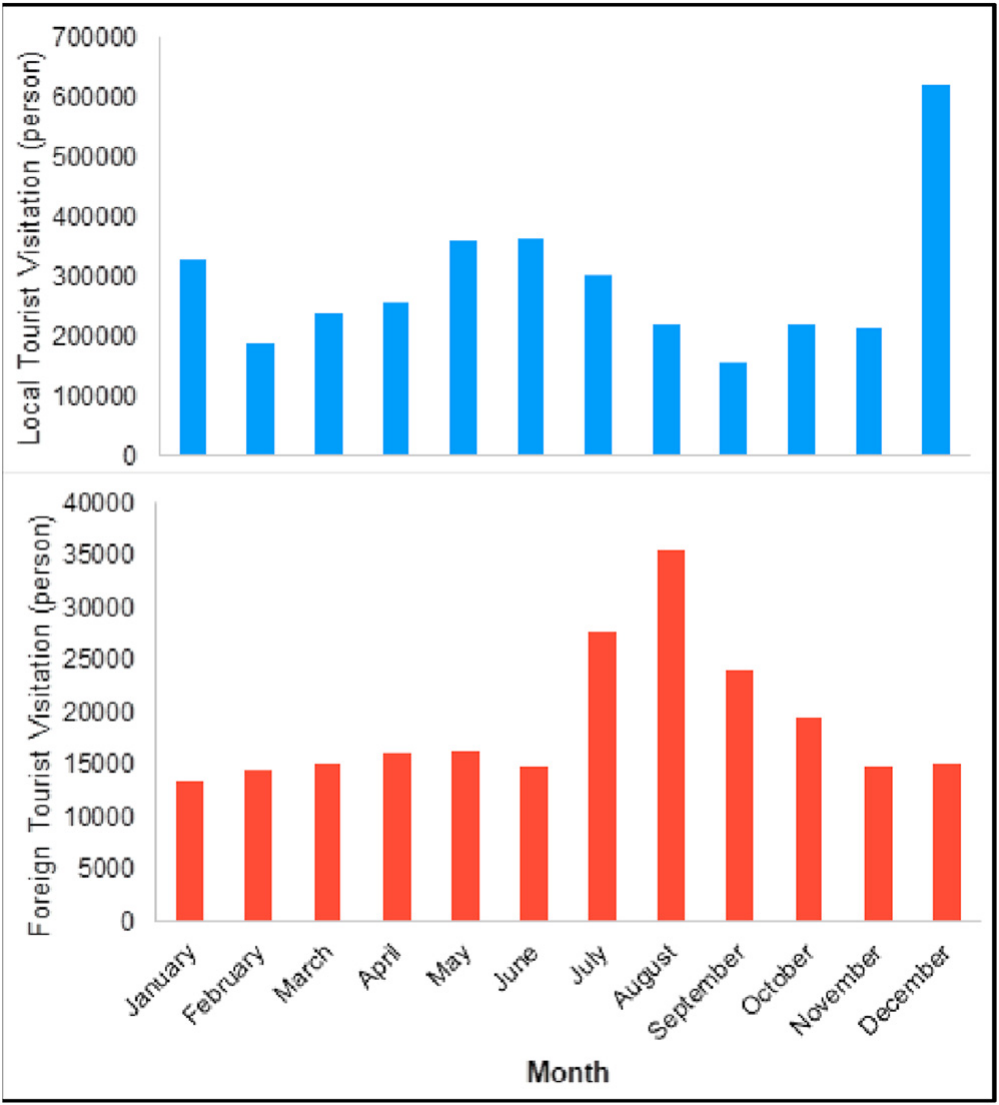
Average monthly tourism visitation in Borobudur Temple in 2013–2019.

Figure 6 shows the number of tourists visiting Borobudur Temple in each month between 2013 and 2019. The figure also shows that domestic tourists far outnumber international ones (concerning to the number of visitors, which is 93.9% of total visitors); this is in lines with the findings of Bigano et al. (2007). According to Pratomo (2017) and Suparwoko (2012), foreign visitors coming to Indonesia are usually drawn to Bali, a major tourist destination because of its tremendous variety of culture and natural richness; by comparison, most of the domestic travelers aim for provinces in Java, including Yogyakarta and the surrounding area, where the Borobudur Temple is located. We can see from the visitation data that August is the month with the most visitors coming from abroad and December is the month with the most domestic tourists. The high number of visitors might indicate the high level of comfort and the temple's suitability for tourism during those particular months. Based on Anđelković et al. (2016), tourists respond to the combined effects of the climate because the human preferences of climate is strongly connected to activities such as tourism.

The results of correlation analysis shown in Table 8 reveal that the TCI and HCI indexes demonstrate a high correlation with foreign tourist visitation data; meanwhile, HCI only has moderate correlation with foreign tourist visitation data. The domestic tourist visitation data only has low correlation with the TCI and HCI, and its correlation with THI is completely negligible. We can see that TCI and HCI are almost at the same level. However, when we calculate the average of the absolute value of each correlation coefficient for both foreign and domestic tourists, we find that HCI is the best index. This proves that our initial hypothesis is correct.

**Table 8:** Correlation rank between tourist visitation data and climate comfort levels from various indexes.

r		x		
		TCI	THI	HCI
y	local	-0.31276	-0.11221	-0.38492
	foreign	0.83392	-0.59981	0.77995
Average of absolute r		0.57334	0.35601	0.58244

The rainfall variable rating system in TCI is not particularly appropriate, because its rainfall-rating scheme is designed in Europe and uses rainfall data from the region; seeing as Europe has relatively low rainfall rates compared to the tropics, the interval which the scheme relies on is relatively small (0–150 mm). The sensitivity test results for HCI and TCI suggest that HCI is more appropriate for studies carried out in tropical areas, which are characterized by relatively high rainfall and high temperatures (Kurnia, 2016).

In this study, we found that foreign tourists are more sensitive to climate comfort than domestic tourists. According to Lohmann and Hübner (2013), the domestic tourists (citizens of a country with warm climate) have already adapted to the conditions and are therefore less dependent on them when choosing a destination. The domestic visitation data usually follows the holiday peak season, as suggested by Haryadi et al. (2019) too. Meanwhile, foreign tourists originating from both warm and cold climate countries take a large set of climate factors into account, including temperature, precipitation, and wind. This is because they need to adapt to the differences between the climate of their own country and that of the visited country.

Tourism depends on the weather, possibly examined by the bioclimatic analysis. Accordingly, the bioclimatic analysis of the temperature and humidity has a primary function in the explication of the climate comfort level shown in the HCI index. Based on Table 9, the relative humidity and precipitation has very high correlation with the climatic comfort level in Borobudur Temple. If we re-check the visitation data (especially foreign tourist visitation), we notice that the number of foreign visitors is high during the dry months (July and August). Foreign tourists prefer nice, dry, sunny summers. Similar with Bae and Nam (2020), in Jeju island, even though the tourist demonstrate that typhoon season is in summer and there is substantial snow in the winter is not a problem. But they prefer sunny weather. Tourists do not enjoy humid climate either. However, Indonesia is one of the Southeast Asia nations that have humid climates.

**Table 9:** Correlation rank between HCI index with its input parameters.

r		x			
		Air temperature max (^o^C)	Relative humidity (%)	Rainfall (mm)	Wind speed (km/hour)
y	HCI	0.36738	-0.71075	-0.71075	0.13639

People's expectations can be a substantial key to understanding the tourists' needs in terms of climatic conditions. Therefore, pre-trip monthly climate comfort information may lead to an increase visitation numbers, as the data could prevent false expectations and help tourists better prepare for their trips. Based on the findings, we can expect that an engineering approach might be needed in and around Borobudur Temple if we want to increase the number of visitors in other months as well. However, outdoor thermal comfort analysis in Borobudur temple in a different engineering approach needs to be explored. As global temperatures are expected to rise, this could be a solution to tackling the negative impact of climate change (Frimpong et al., 2020).

## 4 Conclusion

The comfort conditions in Borobudur Temple occurs differently based on TCI, THI and HCI are July to August, June to August, and July to October, respectively. However, by using a simple analysis method (r), we have managed to establish that the HCI index is the most accurate. In other words, this study proves that HCI is best applied in tropical areas such as the one where the Borobudur Temple is located. Foreign tourists are more sensitive to climate comfort than domestic tourists. The relative humidity and precipitation is highly correlated to the level of climatic comfort. However, neither humidity and precipitation are parameters that can be controlled. People's expectations can be a substantial key to understanding the tourists' needs in terms of climatic conditions. Therefore, pre-trip monthly climate comfort information may lead to an increase in visitation numbers, as the data could prevent false expectations and help tourists better prepare for their trips.

## Declarations

### Author contribution statement

Nur Aini Iswati Hasanah: Conceived and designed the experiments; Performed the experiments; Analyzed and interpreted the data; Contributed reagents, materials, analysis tools or data; Wrote the paper.

Devianti Maryetnowati, Fauziah Nurchaulia Edelweis, Fadhila Indriyani: Performed the experiments; Analyzed and interpreted the data; Contributed reagents, materials, analysis tools or data.

Qorry Nugrahayu: Conceived and designed the experiments; Analyzed and interpreted the data.

### Funding statement

This work was supported by a 2020 grant by the Directorate of Research and Community Services; Universitas Islam Indonesia under the research project entitled “Development of Tourist Climate Comfort Information on Halal Tourism Destinations in Yogyakarta and Surrounding Areas” No: 09/Dir/DPPM/70/Pen.Pemula/III/2020.

### Data availability statement

Data will be made available on request.

### Declaration of interests statement

The authors declare no conflict of interest.

### Additional information

No additional information is available for this paper.
